# A Study of Hydroxyl-Terminated Block Copolyether-Based Binder Curing Kinetics

**DOI:** 10.3390/polym16162246

**Published:** 2024-08-07

**Authors:** Wu Yang, Zhengmao Ding, Cong Zhu, Tianqi Li, Wenhao Liu, Yunjun Luo

**Affiliations:** 1 School of Materials Science and Engineering, Beijing Institute of Technology, Beijing 100081, China; 2 Pen-Tung Sah Institute of Micro-Nano Science and Technology, Xiamen University, Xiamen 361005, China

**Keywords:** HTPE, curing kinetics, non-isothermal DSC, isoconversional methods, autocatalytic reaction model

## Abstract

In order to determine the curing reaction model and corresponding parameters of hydroxyl-terminated block copolyether (HTPE) and provide a theoretical reference for its practical application, the non-isothermal differential scanning calorimetry (DSC) method was used to analyze the curing processes of three curing systems with HTPE and N-100 (an aliphatic polyisocyanate curing agent), isophorone diisocyanate (IPDI), and a mixture of N-100 and IPDI as curing agents. The results show that the curing activation energy of N-100 and HTPE was about 69.37 kJ/mol, slightly lower than the curing activation energy of IPDI and HTPE (75.60 kJ/mol), and the curing activation energy of the mixed curing agent and HTPE was 69.79 kJ/mol. The curing process of HTPE conformed to the autocatalytic reaction model. The non-catalytic reaction order (n) of N-100 and HTPE was about 1.2, and the autocatalytic order (m) was about 0.3, both lower than those of IPDI and HTPE. The reaction kinetics parameters of the N-100 and IPDI mixed curing agent with HTPE were close to those of N-100 and HTPE. The verification results indicate a high degree of overlap between the experimental data and the calculated data.

## 1. Introduction

Hydroxyl-terminated block copolyether (HTPE), as a block copolymer ether composed of polyethylene glycol (PEG) and polytetrafluoroethylene (PTMG), is considered to be a new type of binder that is expected to replace hydroxyl-terminated polybutadiene (HTPB) in the field of composite solid propellants [1] due to its excellent performance, particularly its outstanding low vulnerability characteristics [2]. At present, a large number of works on the mechanics [3,4], thermal decomposition [5,6], combustion [7,8,9], and safety performance [10,11] of HTPE-based propellants have been published, and numerous studies have shown that HTPE has good application prospects in the field of composite solid propellants.

However, there are few reports on the curing kinetics of HTPE. The curing reaction between HTPE and the curing agent is directly related to the process parameters, such as the pot life and curing time of the propellant, and is an important step in the preparation process of composite solid propellants [12]. Therefore, studying the curing kinetics of HTPE helps to better understand its curing reaction process and is very helpful for the specific application of HTPE [13].

Many tests can be used to analyze the curing kinetics, such as low-field nuclear magnetic resonance testing [14], rheological testing [15,16], infrared testing [17,18], differential scanning calorimetry (DSC) testing [19,20,21], etc. Among them, the DSC method has the characteristics of high sensitivity, simple operation, and a short testing cycle and has been widely used. DSC testing can detect and record the heat changes during the curing reaction process. By mathematically processing and analyzing the recorded data, researchers can obtain the reaction model and kinetic parameters of the curing reaction [22]. It should be noted that there are two commonly used methods for the study of curing kinetics parameters using DSC, namely the isothermal method and the non-isothermal method. These two methods each have their own characteristics and scope. Among them, the isothermal method tests the curing reaction of a sample at multiple fixed temperatures (usually at least four different temperatures), records the change in heat release during the curing reaction process with the reaction time until the end of the reaction, and analyzes it. This method can test the curing reaction of the sample at ambient temperature during its actual application, which is more in line with the actual application situation of the sample. However, its disadvantage is that when the curing reaction of the sample is slow and the heat released during the reaction is low, it can lead to a long testing cycle and the inaccurate detection of the instrument in the later stage of the curing reaction due to the low heat generated by the reaction, resulting in serious data errors. This is unacceptable for the analysis of curing reaction kinetics. Therefore, the isothermal DSC method is commonly used to study reactions with high heat release and fast reaction rates, such as photopolymerization reactions. However, the curing reaction of HTPE, studied in this work, does not meet the requirements. On the other hand, the non-isothermal DSC method records the variation in the heat release of the sample during the curing reaction with the temperature at different heating rates (usually at least four different heating rates) within a certain temperature range, until the curing reaction is complete, and analyzes it. Compared to the isothermal DSC method, the non-isothermal DSC method has a shorter testing period and more accurate data, making it more commonly used in reaction kinetics analysis. This is also the method applied in this study. It is worth noting that whether using the isothermal DSC method or the non-isothermal DSC method, it should be ensured that only the curing reaction of the sample produces thermal effects during the testing process, and there are no other processes (such as volatilization, thermal decomposition, etc.), because the use of the DSC method to study the curing reaction kinetics is based on the reaction heat data recorded by DSC. Otherwise, it will result in inaccuracies or the impossibility of analyzing the curing kinetics. Therefore, it is necessary to choose an appropriate temperature or heating rate.

Moreover, the principle of the HTPE curing reaction is that the hydroxyl groups at the ends of the molecular chains of the HTPE prepolymer react with the isocyanate groups in the curing agent to form amino ester groups. During the reaction, the molecular chains expand to form a three-dimensional crosslinked network, transforming the reactants from a liquid to a solid state. Therefore, the curing reaction of HTPE requires the participation of curing agents with isocyanate groups and a minimum functionality of 2; otherwise, the expansion of the molecular chains cannot be achieved. There are many types of curing agents that meet these requirements, of which N-100 (an aliphatic polyisocyanate curing agent) is a commonly used curing agent for the curing reaction of HTPE due to its functionality being greater than 3, making it easy to form chemical crosslinking points [23,24,25]. On the other hand, isophorone diisocyanate (IPDI) is a commonly used curing agent for polyurethane curing reactions [26,27], and it has also been applied in the HTPE system [28]. Therefore, in this study, to determine the reaction model and corresponding kinetic parameters of the HTPE curing reaction, and to provide certain references for the practical application of HTPE, the non-isothermal DSC method was used to analyze the curing kinetics of HTPE with N-100, IPDI, and mixtures of N-100 and IPDI. Finally, it was found that the curing reaction process of HTPE with N-100 and IPDI was consistent with the autocatalytic reaction model, and the corresponding kinetic parameters were obtained by calculation and fitting.

## 2. Materials and Methods

### 2.1. Materials

Hydroxyl-terminated block copolyether (HTPE) and N-100 (an aliphatic polyisocyanate curing agent) were supplied by the Liming Research Institute of Chemical Industry, Luoyang, China. Isophorone diisocyanate (IPDI) was obtained from Aladdin. Dioctyl sebacate (DOS) was analytically pure and obtained from the Tianjin Guangfu Fine Chemical Research Institute. Triphenyl bismuth (TPB) (purity of 99%) and dibutyltin dilaurate (DBTDL) were obtained from the Shanghai Institute of Organic Chemistry (Shanghai Municipality, China) and were formulated into a 0.5 wt.% solution with DOS as the solvent and mixed in a mass ratio of TPB/DBTDL of 3:1 before being used as the curing catalyst [29].

### 2.2. Non-Isothermal Differential Scanning Calorimetry Tests

Non-isothermal differential scanning calorimetric (DSC) analysis was carried out in the DSC 3 (METTLER TOLEDO). Approximately 5~8 mg of the reaction mixture, comprising HTPE and the curing agent at a stoichiometric ratio of 1, was placed in an aluminum crucible. Before placing the mixture into the crucible, it needed to be thoroughly stirred to ensure that the curing agent, catalyst, and binder were mixed evenly. Then, the mixture was subjected to a heating schedule up to 473 K from 298 K at various heating rates (1, 2, 3, and 4 K/min) under an atmosphere of nitrogen, purged at a rate of 40 mL/min. The curing agents used were N-100, IPDI, and a mixed curing agent composed of N-100 and IPDI. According to previous experiments, when the mass ratio of N-100 to IPDI was 4:1, the prepared adhesive had good performance. Therefore, in the curing kinetics study in this work, the mass ratio of N-100 to IPDI contained in the mixed curing agent used was 4:1. The curing system was named N-100/HTPE, IPDI/HTPE, or NI/HTPE according to the curing agent used.

## 3. Results

### 3.1. Curing Activation Energy

**Figure 1:**
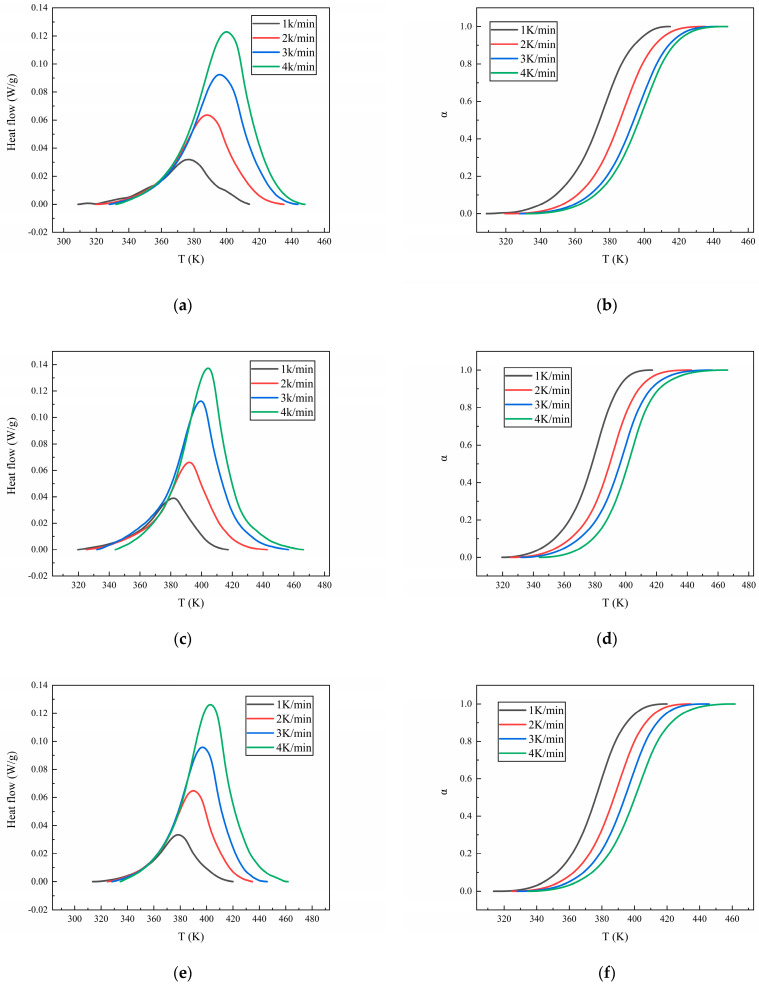
(**a**) The heat flow curves of N-100/HTPE, (**b**) the α versus temperature curves of N-100/HTPE, (**c**) the heat flow curves of IPDI/HTPE, (**d**) the α versus temperature curves of IPDI/HTPE, (**e**) the heat flow curves of NI/HTPE, and (**f**) the α versus temperature curves of NI/HTPE. The colors of the curves in each graph represent the heating rates, with black, red, blue, and green representing heating rates of 1 K/min, 2 K/min, 3 K/min, and 4K/min, respectively.

The heat flow curves of N-100/HTPE, IPDI/HTPE, and NI/HTPE are shown in Figure 1, and the corresponding conversion rate (α) versus temperature curves are also displayed. It is worth noting that different testing methods correspond to different calculation methods for the conversion rates. For non-isothermal DSC methods, calculating the conversion rates requires the integration of the heat flow curves at different heating rates to obtain the total heat release of the curing reaction at each heating rate. Then, the accumulated heat release at a certain time during the reaction process can be compared with the total heat release of the entire reaction to obtain the conversion rate α at that time. At the beginning of the reaction, α is 0, while, at the end of the reaction, α is 1. This is because, when analyzing the reaction kinetics using the DSC method, it is believed that only the reactants will produce thermal effects when they undergo a reaction—that is, the beginning and end of the exothermic reaction are used as indicators of the start and end of the reaction, and the exothermic process represents the reaction process.

From Figure 1, it can be seen that the heat flow curves of the three curing systems all have only one exothermic peak. As the heating rate increases, the peak temperature (*T*_max_) also increases, and the shape of the exothermic peak remains almost unchanged but becomes higher and sharper. This is a common phenomenon in non-isothermal DSC testing analysis. The reason is that, in the non-isothermal DSC testing process, the test sample is heated by the instrument, and the heating rate is controlled by the program. However, in reality, the temperature distribution inside the sample is uneven. The faster the heating rate, the more uneven the temperature distribution inside the sample and the greater the temperature difference between the inside and outside, which leads to an increase in the peak temperature. At the same time, an increase in the heating rate will also cause the sample to reach a higher temperature faster, resulting in an increase in the peak temperature with an increase in the heating rate. Moreover, an increase in the heating rate will lead to an increase in the thermal effect per unit of time, resulting in greater temperature changes and an increase in the peak height. Meanwhile, the conversion rates of the three curing systems exhibit a typical S-shaped curve as a function of the temperature, and the slope of the curve increases with the increase in the heating rate.

**Figure 2:**
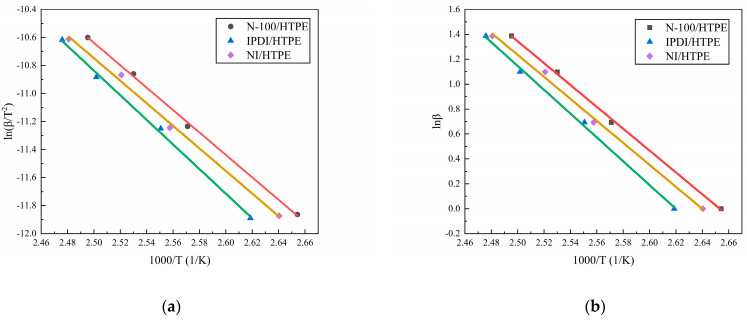
(**a**) The fitting results of the activation energy for the three curing systems using the Kissinger method and (**b**) the fitting results of the activation energy for the three curing systems using the Ozawa method. The dots in the figure represent the experimental data, the lines represent the fitting results, and the colors of the lines represent the different curing systems, where red represents N-100/HTPE, green represents IPDI/HTPE, and brown represents NI/HTPE.

Equations (1) and (2) represent the Kissinger model [30] and the Ozawa model [31], respectively. It can be seen from them that the curing activation energy (*E*_a_) can be calculated based on the *T*_max_ of the heat flow curves at different heating rates. The *T*_max_ and activation energies of the three curing systems at different heating rates are listed in Table 1. Figure 2 shows the fitting results of the three curing systems with the Kissinger and Ozawa models.

(1) $$\frac{d\ln\left(\beta /T_{\max}^{2}\right)}{d\left(1/T_{\max}\right)}=\;-\frac{E_{a}}{R}$$

(2) $$\frac{d\ln\beta }{d\left(1/T_{\max}\right)}=\;-1.052\frac{E_{a}}{R}$$

where β is the heating rate, in K/min; R is the gas constant, with a value of 8.314 J/(mol·K).

Table 1 shows that the order of the peak temperatures for the three curing systems at various heating rates is N-100/HTPE < NI/HTPE < IPDI/HTPE, and the order of the activation energies is also the same. This indicates that, compared to IPDI, N-100 is more likely to react with HTPE, but the difference in reaction activity between the two curing agents is very minimal. From Figure 1, it can also be seen that, like N-100/HTPE and IPDI/HTPE, NI/HTPE only has one exothermic peak, and it cannot be divided into two or more through fitting. Therefore, it is reasonable to believe that both the reaction between N-100 and HTPE and the reaction between IPDI and HTPE occur simultaneously during the curing of the NI/HTPE system. It should be noted that the activation energies calculated using the Kissinger method and Ozara method are fixed values, which are not accurate enough and only serve as a reference, since the activation energy of the curing reaction is constantly changing with the progress of the reaction in reality.

**Figure 3:**
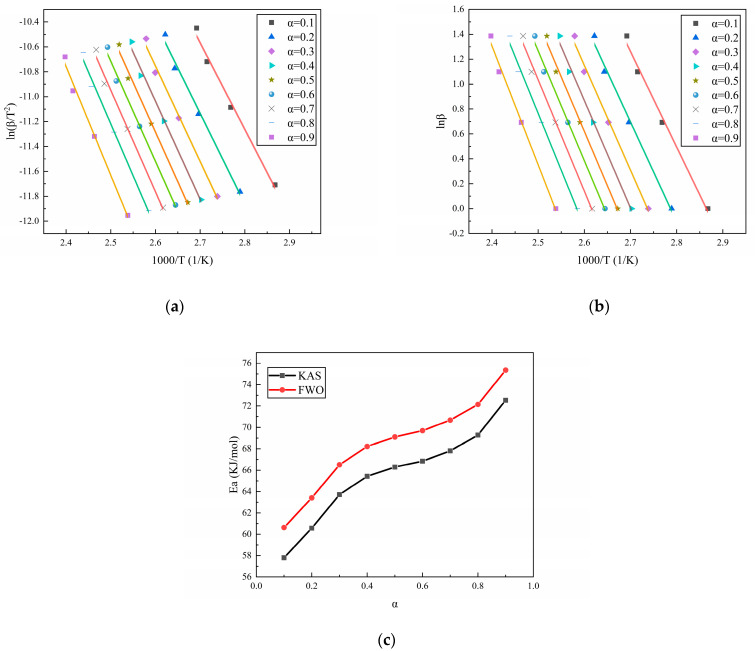
(**a**) The fitting results of N-100/HTPE based on the KAS method, (**b**) the fitting results of N-100/HTPE based on the FWO method, and (**c**) the activation energy calculation results of N-100/HTPE based on the KAS and FWO methods.

**Figure 4:**
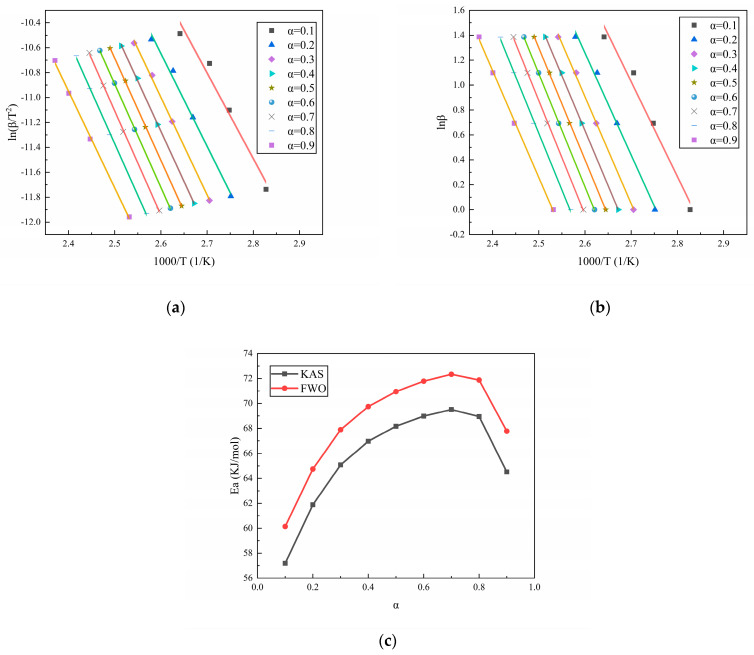
(**a**) The fitting results of IPDI/HTPE based on the KAS method, (**b**) the fitting results of IPDI/HTPE based on the FWO method, and (**c**) the activation energy calculation results of IPDI/HTPE based on the KAS and FWO methods.

**Figure 5:**
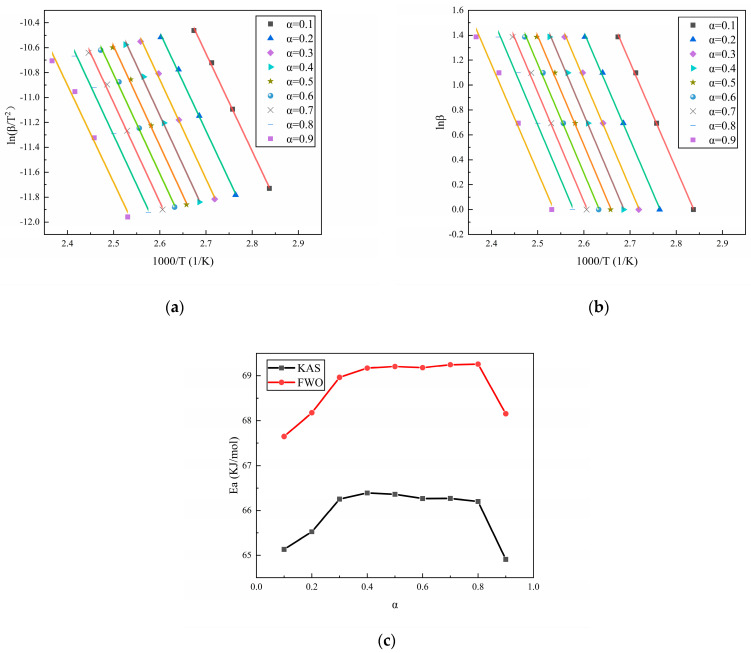
(**a**) The fitting results of NI/HTPE based on the KAS method, (**b**) the fitting results of NI/HTPE based on the FWO method, and (**c**) the activation energy calculation results of NI/HTPE based on the KAS and FWO methods.

The isoconversional method is a commonly used method for the calculation of the change in activation energy during the reaction process. Among them, isoconversional methods such as the Kissinger–Akahira–Sunose (KAS) method [32] and Flynn–Wall–Ozawa (FWO) method [33], as extensions of the Kissinger method and Ozawa method, respectively, are also commonly used to study the changes in activation energy during the reaction process. According to the conversion rate–temperature curves of the three curing systems at different heating rates, as displayed in Figure 1, the activation energy during the reaction process can be calculated using the isoconversional method. Figure 3, Figure 4 and Figure 5 show the calculation results of the activation energies of N-100/HTPE, IPDI/HTPE, and NI/HTPE using the KAS method and FWO method, respectively.

From Figure 3, Figure 4 and Figure 5, it can be seen that the changes in the activation energies calculated by the KAS and FWO methods have the same trends, and although the activation energies calculated by the KAS and FWO methods are different, the difference is very small. Similarly, the activation energies calculated by the Kissinger and Ozawa methods in Table 1 are also minimal. This is understandable as the KAS and FWO methods are extensions of the Kissinger and Ozawa methods, respectively. Furthermore, it can be observed that the activation energy changes of the IPDI/HTPE system are similar to those in reference [34], indicating that there is a significant diffusion control process in the later stage of the curing reaction. Through comparison, it can be found that the NI/HTPE system also exhibits similar phenomena, which should be attributed to the role of IPDI in the curing agent. On the other hand, as the reaction progresses, the concentrations of the reactants decrease, resulting in a decrease in the effective collision frequency between the reactants. This is also one of the reasons that the activation energy increases as the reaction progresses.

### 3.2. Curing Kinetics Model and Parameters

**Figure 6:**
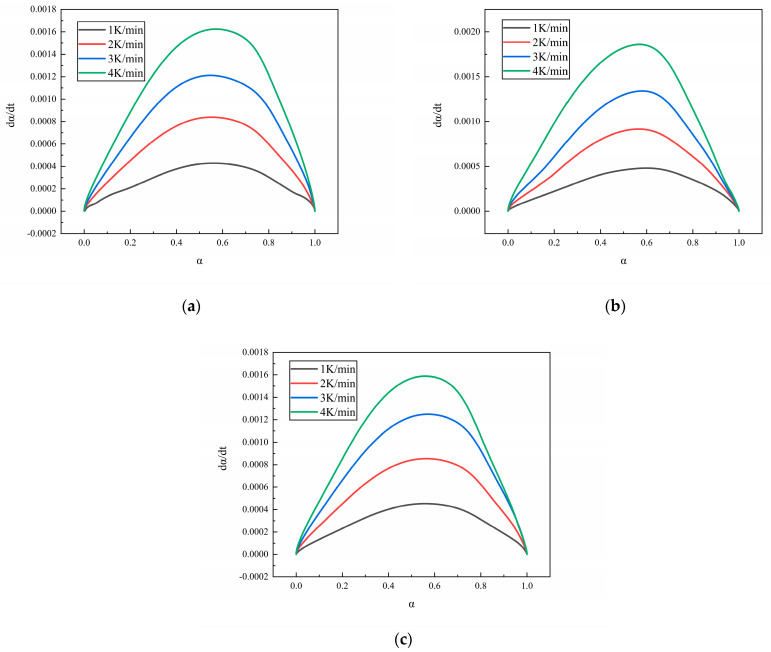
(**a**) The reaction rate dα/dt versus α curves of N-100/HTPE, (**b**) the reaction rate dα/dt versus α curves of IPDI/HTPE, and (**c**) the reaction rate dα/dt versus α curves of NI/HTPE. The colors of the curves in each graph represent the heating rates, with black, red, blue, and green representing heating rates of 1 K/min, 2 K/min, 3 K/min, and 4/min, respectively.

Figure 6 shows the curves of dα/dt versus α for the N-100/HTPE, IPDI/HTPE, and NI/HTPE systems, all of which exhibit the characteristics of autocatalytic reactions [35,36]. In addition, some references also show that the reaction of a hydroxy-terminated polymer with isocyanate is autocatalytic [13,37,38,39]. Based on this, it is reasonable to assume that the reactions of these three systems are all autocatalytic reactions.

Equation (3) shows the autocatalytic reaction model [32], where m is the order of the autocatalytic reaction, n is the order of the non-catalytic reaction, and A is the pre-exponential factor. Equation (3) can be easily transformed to obtain Equation (4), and replacing α with (1 − α) in Equation (4) gives Equation (5), while adding or subtracting Equation (4) from Equation (5) gives Equations (6) and (7) [40]. From Equations (6) and (7), it can be seen that by linearly fitting the scatter plot of Value I to ln((1 − α)/α), the slope obtained is n − m. By linearly fitting the scatter plot of Value II to ln(α − α^2^), the slope obtained is n + m, and the intercept is 2lnA. Further calculation can obtain the values of m, n, and A.

(3) $$\frac{d\alpha }{\mathrm{dt}}=\mathrm{Aexp}\left(-\frac{E_{a}}{RT}\right)\alpha^{m}{\left(1-\alpha \right)}^{n}$$

(4) $$\ln\left(\frac{d\alpha }{\mathrm{dt}}\right)=\ln A-\frac{E_{a}}{RT}+m\ln\alpha +n\ln\left(1-\alpha \right)$$

(5) $$\ln\left(\frac{d\left(1-\alpha \right)}{\mathrm{dt}}\right)=\ln A-\frac{E_{a}}{RT^{\prime }}+m\ln\left(1-\alpha \right)+n\ln\alpha$$

(6) $$\mathrm{Value}\;I=\ln\left(\frac{d\alpha }{\mathrm{dt}}\right)+\frac{E_{a}}{RT}-\ln\left[\frac{d\left(1-\alpha \right)}{\mathrm{dt}}\right]-\frac{E_{a}}{RT^{\prime }}=\left(n-m\right)\ln\left(\frac{1-\alpha }{\alpha }\right)$$

(7) $$\mathrm{Value}\;\mathrm{II}=\ln\left(\frac{d\alpha }{\mathrm{dt}}\right)+\frac{E_{a}}{RT}+\ln\left[\frac{d\left(1-\alpha \right)}{\mathrm{dt}}\right]+\frac{E_{a}}{RT^{\prime }}=\left(n+m\right)\ln\left(\alpha -\alpha^{2}\right)+2\ln A$$

**Figure 7:**
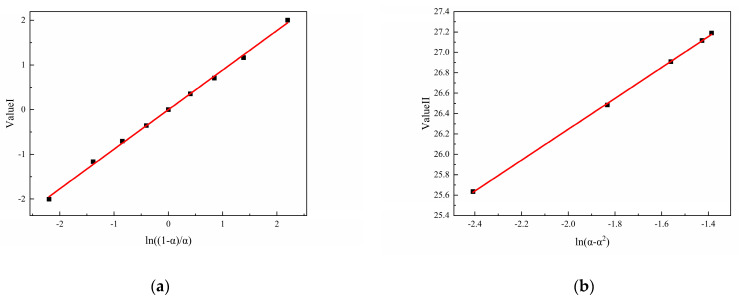
(**a**) The typical plots of Value I calculated using DSC data for N-100/HTPE and (**b**) the typical plots of Value II calculated using DSC data for N-100/HTPE.

**Figure 8:**
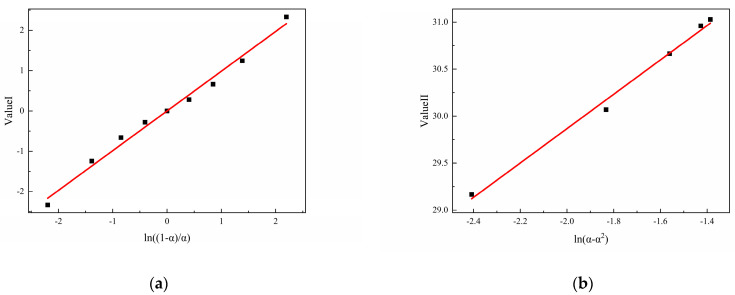
(**a**) The typical plots of Value I calculated using DSC data for IPDI/HTPE and (**b**) the typical plots of Value II calculated using DSC data for IPDI/HTPE.

**Figure 9:**
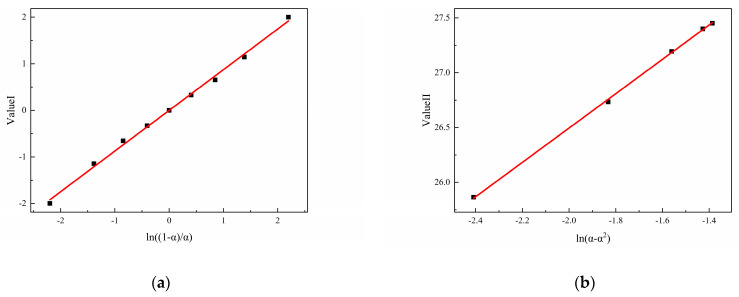
(**a**) The typical plots of Value I calculated using DSC data for NI/HTPE and (**b**) the typical plots of Value II calculated using DSC data for NI/HTPE.

Assuming that the curing reactions of N-100/HTPE, IPDI/HTPE, and NI/HTPE follow the autocatalytic reaction model, according to Equations (6) and (7), it can be seen that Value I and Value II, obtained by the mathematical processing of the experimental data of the three curing systems, should have good linear relationships with ln((1 − α)/α) and ln(α − α^2^), respectively. This is a method that can be used to verify whether the curing reaction conforms to the autocatalytic reaction model. Based on this, the experimental data of the three curing systems were mathematically processed according to Equations (6) and (7) and plotted and linearly fitted. The results are shown in Figure 7, Figure 8 and Figure 9.

From Figure 7, Figure 8 and Figure 9, it can be seen that Value I and Value II of the three curing systems all have a relatively strong linear correlation with the corresponding ln((1 − α)/α) and ln(α − α^2^), indicating that the curing reactions of the three curing systems are all in line with the autocatalytic reaction model. Meanwhile, based on the linear fitting results, the corresponding kinetic parameters (m, n, and A) can be calculated and are listed in Table 2. On the other hand, after confirming that the reaction conforms to the autocatalytic reaction model, the experimental data were fed into Equation (3) and fitted using the Auto2Fit software (Professional Version 5.5) to obtain the corresponding kinetic parameters, which are also listed in Table 2.

From Table 2, it is evident that the kinetic parameters calculated using Equations (6) and (7) closely match the kinetic parameters obtained through the Auto2Fit software, suggesting the accuracy of the obtained kinetic parameters. Through comparison, it can be found that the autocatalytic order m of the three curing systems is smaller than the non-catalytic reaction order n, indicating that although the reactions of the three systems conform to the autocatalytic reaction model, the autocatalytic effect does not play a dominant role in the reaction process, which is also similar to related research [15,16,37,38,41]. This may partly explain why the activation energy increases as the conversion rate during the reaction process rises. Furthermore, it can be observed that the kinetic parameters of N-100/HTPE are lower than those of IPDI/HTPE, while the kinetic parameters of NI/HTPE are slightly higher than those of N-100/HTPE. Given that the curing agent used in the NI/HTPE system is a mixture of N-100 and IPDI, most of which is N-100, this phenomenon is understandable. In addition, it can be noted that the total reaction orders (m + n) of the three curing systems are all greater than 1 and less than 2, which is also normal because the total reaction orders of almost all polyurethane curing reactions are within this range of 1 to 2, indicating that the calculated reaction order parameters are reasonable.

### 3.3. Model Validation

**Figure 10:**
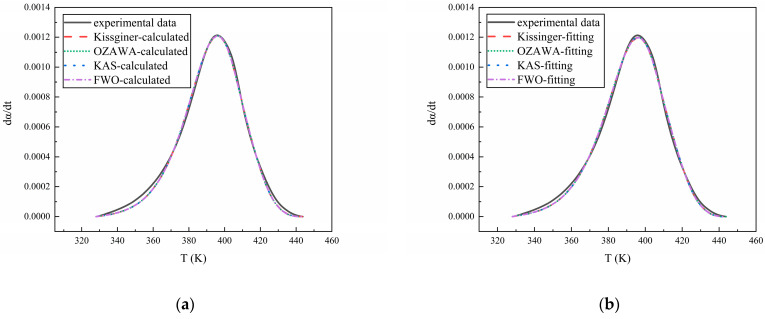
(**a**) The comparison of the experimental data of N-100/HTPE with the calculated results and (**b**) the comparison of the experimental data of N-100/HTPE with the fitting results.

**Figure 11:**
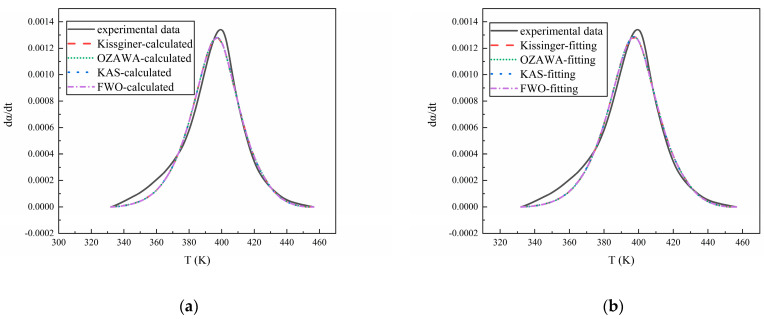
(**a**) The comparison of the experimental data of IPDI/HTPE with the calculated results and (**b**) the comparison of the experimental data of IPDI/HTPE with the fitting results.

**Figure 12:**
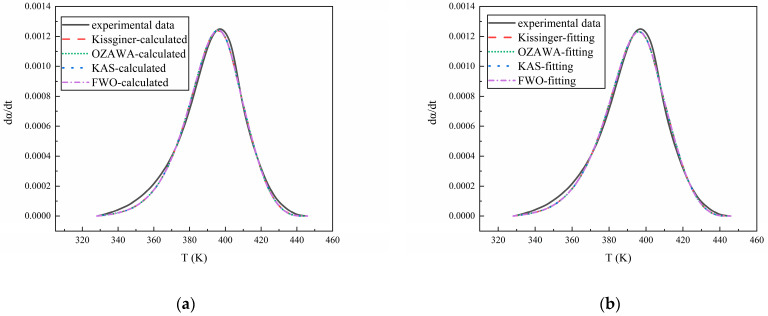
(**a**) The comparison of the experimental data of NI/HTPE with the calculated results and (**b**) the comparison of the experimental data of NI/HTPE with the fitting results.

Finally, by inputting the kinetic parameters from Table 2 into Equation (3), the curve of the dα/dt versus temperature for each curing system can be obtained. By comparing it with the experimental data, the accuracy of the reaction model and kinetic parameters can be verified. The results are shown in Figure 10, Figure 11 and Figure 12, from which it can be seen that the calculated results and the fitted results agree well with the experimental curve, proving the validity of the reaction kinetics model and related parameters.

## 4. Conclusions

The activation energy of the reaction between N-100 and HTPE is 69.37 kJ/mol, slightly lower than that of the reaction between IPDI and HTPE (75.60 kJ/mol), and the curing activation energy of the mixed curing agent and HTPE is 69.79 kJ/mol. However, when N-100 and IPDI are mixed as curing agents, both curing agents should react with HTPE at the same time, without a clear sequence. In addition, it has been proven through both calculation and fitting that the curing reactions of the N-100/HTPE, IPDI/HTPE, and NI/HTPE systems all conform to the autocatalytic reaction model. The non-catalytic reaction order n of N-100/HTPE is about 1.2, and the autocatalytic order m is about 0.3, both of which are lower than those of IPDI/HTPE, while the non-catalytic reaction order and autocatalytic order of NI/HTPE are between those of N-100/HTPE and IPDI/HTPE. Model validation shows that the obtained model and corresponding parameters have high accuracy. The research results of the curing kinetics obtained in this work contribute to a better understanding of the curing process of HTPE and provide guidance for its practical application.

## Figures and Tables

**Table 1:** *T*_max_ and *E*_a_ of the three curing systems.

Curing System	Peak Temperature *T*_max_ (K)				*E*_a_ (kJ/mol)	
	1 K/min	2 K/min	3 K/min	4 K/min	Kissinger	Ozawa
N-100/HTPE	376.753	388.952	395.251	400.745	66.53	69.37
IPDI/HTPE	381.872	392.055	399.711	403.886	73.01	75.60
NI/HTPE	378.736	390.987	396.699	403.084	66.94	69.79

**Table 2:** The curing kinetic parameters of the three curing systems obtained through calculation and fitting.

Designation			N-100/HTPE	IPDI/HTPE	NI/HTPE
m	Kissinger	calculated	0.314	0.421	0.347
		fitting	0.274	0.456	0.311
	Ozawa	calculated	0.282	0.391	0.315
		fitting	0.242	0.428	0.279
	KAS	calculated	0.325	0.506	0.358
		fitting	0.284	0.535	0.322
	FWO	calculated	0.293	0.472	0.326
		fitting	0.252	0.504	0.290
n	Kissinger	calculated	1.200	1.407	1.220
		fitting	1.156	1.409	1.176
	Ozawa	calculated	1.214	1.419	1.234
		fitting	1.170	1.422	1.190
	KAS	calculated	1.195	1.373	1.215
		fitting	1.151	1.371	1.172
	FWO	calculated	1.209	1.386	1.229
		fitting	1.165	1.386	1.185
lnA	Kissinger	calculated	14.64	16.76	14.82
		fitting	14.57	16.79	14.75
	Ozawa	calculated	15.49	17.54	15.67
		fitting	15.42	17.57	15.61
	KAS	calculated	14.35	14.58	14.51
		fitting	14.29	14.60	14.44
	FWO	calculated	15.20	15.44	15.37
		fitting	15.14	15.46	15.30

## Data Availability

Data are contained within the article.
